# Malaria, with Cases and Remarks on Renal Complication

**Published:** 1880-11

**Authors:** I. M. Clemens


### Selections.

### MALARIA, WITH CASES AND REMARKS ON RENAL COMPLICATION.

By I. M. CLEMENS, M.D.

"No medical question is of comparable importance to that of malaria to the people of the United States; nay, to the world. It is less virulent and abundant in some of the older and better drained parts of this country and of Europe than it is in others, but wherever the sun shines and water exists malaria will sometimes be found. It is almost omnipresent, and for evil almost omnipotent."—*Prof. L. P. Yandell to C. H. Lathrop, Lyons, Iowa.*

No medical truth comparable in importance with the above to the people of the United States has been enunciated in this the nineteenth century. We need not pause to discuss the question, "What is malaria?" The whether we are able, in the light of the present advanced stage of scientific inquiry, to demonstrate it satisfactorily matters not for the purposes of this article. With the conditions necessary to the production of malaria in the common acceptation of the term every medical man is familiar. Not every medical man, however, appears to be impressed with the potency and protean manifestations of this ubiquitous enemy to health and life.

If our National Board of Health do no more than awaken States, municipalities, and citizens to a realizing sense of the invisible danger that lurks everywhere, in the filthy gutter, the choked sewer, the dirty alley and back yard, the foul waste pipe, the damp cellar, the damp, moldy wall, ill-ventilated houses—point out and secure the adoption and enforcement of the means to obviate it, it will have immortalized itself. The people should be educated to know their danger from these sources, that they may contribute to the general welfare of their neighborhoods by putting their own premises in proper sanitary condition.

Hundreds of little children are sacrificed to this insatiable Moloch every summer and fall in this and every other similarly filthy Southern and Western city, by flocking to the sidewalks at nightfall to get a breath of cool air, often stringing themselves along the curbstone, like so many birds, over the filthy gutters and near the mouths of foul sewers, they are immersed in an atmosphere only a little less deadly than that emenating from the terrible Pontaine marshes in Italy.

So also every night hundreds of families in this city are shut up in old brick houses, with brick foundations which have absorbed moisture from the earth for years until they have become rotten, so to speak, from long-continued dampness, the plaster and brick in many cases absolutely rotten and crumbling away. All unsuspectingly they are breathing an atmosphere only a little less deadly than that of the historic Black Hole of Calcutta. The inevitable consequence is sometimes manifested in a comparatively slight indisposition, but often in the taking off of some member of the family with bewildering swiftness. Such houses are a curse to the city, and in the interest of the public health should be condemned to be razed to the earth, or, in case of the better class of them, to have a layer of window glass placed in the wall at a few inches above ground to intercept the moisture.

For the safety of surrounding property municipal governments arrogate to themselves the power to pass and enforce ordinances declaring that within certain limits no frame building shall be erected. Why may they not, for the safety of the public health, require that every house to be built with a brick foundation shall have, at a suitable elevation above ground, a layer of window-glass carefully placed, end to end, and of a width corresponding to that of the wall? From three to ten dollars, according to the size of the house, would forever protect it against the moisture, which is drawn up through the porous bricks and plaster by capillary attraction, and make the houses perfectly dry for all time, as the glass is absolutely indestructable by the agencies to which it would be subjected.

It will be observed that I have not mentionod ponds, marshes, the banks of the sluggish streams, etc., which are understood by the people to be the common and by many the sole sources of malarial poison, but have attempted rather to make conspicuous those sources especially in cities to which the people as a mass seem to be oblivious, and with the fruitfulness and danger of which I am led to believe many physicians are not sufficiently impressed.

As illustrating the potency of the malarial poison generated in the class of houses alluded to above, I wish to mention three cases of what may be termed unclassified malarial fever which I have recently met with in one of these houses with otherwise healthy surroundings. I wish also to call attention to a renal complication, a feature I have observed in a number of similar cases of profound malarial poisoning under similar circumstances in this city in the last two or three years.

About eight o'clock on the evening of August 29, 1880, I was called to see two children of L. K., aged respectively four and a half and seven and a half years. They had previously been in good health, and without prodromic symptoms, so far as the parents knew, were observed to have high fever in the afternoon four or five hours prior to my visit. Under the influence of a warm bath the younger of the two, a little girl, was just emerging from a violent convulsion, which had lasted for more than half an hour. She had some nausea and had vomited two or three times during the afternoon and evening; bowels regular; tongue somewhat enlarged and covered by a uniform white coat of moderate thickness. The breath was of that peculiarly offensive character noticeable in zymotic diseases, resembling most that of scarlatina. The conjunctivæ were somewhat injected and the pupils slightly contracted, but not over-sensitive to light. After fully recovering from the convulsion her intelligence seemed to be good. The thermometer placed in the axilla marked 107°; pulse 150; skin moist; face pale, considering the extreme high temperature.

The boy's case was the counterpart of this, including temperature, with the following exceptions: Pupils were normal; pulse 140; had not vomited or had a convulsion. Notwithstanding the absence of the usually flushed face and surface and strawberry tongue, the pulse, temperature and nervous phenomena, together with the fact that scarlatina was known to exist in that region of the city, suggested the strong probability that I had this disease to deal with. The mother having administered to each of them in the afternoon a dose of castor oil, I ordered potassa bromidi with fluid ext. gelsemium to control the nervous symptoms; a febrifuge mixture of liq. ammonia acetatis, spts. eth. nit., and tinct. aconite rad., together with a tepid sponge-bath in case the skin should become dry.

On the 30th, at 9:30 A.M., I found them in much the same condition (temperature same), except that their bowels had been moved; the little girl having had a convulsion in the afterpart of the night and one prior to my visit in the morning. The day-time affording more hope that the medicine would be faithfully administered, I ordered a continuance of the remedies prescribed the previous evening, with the addition of chloral hydrate for the little girl when a convulsion seemed imminent. At 3:30 P.M. I found them again without material change in their condition; the boy more nervous, slight tendency to delirium, pulse 148; the girl's pulse 168, convulsions more frequent, with marked opisthotonus.

By exclusion and in the light of the surroundings I was now convinced that I had to deal with an intense malarial poison, and without waiting longer for a remission I put them on full doses of quinia sulphate every two hours. Through fear that the girl would be unable to take a sufficiency per orem, I ordered twenty grains with ten minims acid sulph. arom., rubbed up with half an ounce of vaseline, one-fourth to be rubbed into the epigastric and hypochondriac regions every three hours.

At 10:30 P.M. I found the girl, to whom they had been unable to administer the medicine with any satisfaction, in a persistent eclampsia; in other respects the same, except the pulse, which had less volume. The boy's general condition was somewhat improved; pulse 140, temperature 105 deg. I remained with them some hours, sponging the girl persistently, and finally putting her for thirty minutes in a bath at 100 deg., which reduced the temperature to 105 deg. and for half an hour rendered her more quiet. At the end of which time the former elevation of temperature was regained and the eclampsia returned.

At 1:30 A.M. the boy's temperature had descended to 103°. He was sleeping and in a profuse perpiration. The girl was ordered to be bathed and sponged by turns, in the hope of reducing the temperature. This did not avail, however. The opisthotonus became persistent, and she died at 4:30 A.M. At 9:30 the following morning the boy's temperature was 101°; pulse 100, with corresponding improvement in all his symptoms. At 5 P.M. temperature 100°, pulse 90. On the following morning the temperature was normal. and in the afternoon he was dressed and playing in the bed.

On the afternoon of the 31st, the day on which the girl died, my attention was called to the mother—an ordinarily stout, healthy-looking German woman, aged about thirty-three—who stated that she had felt a little cold on the afternoon of the day previous, and following; had a fever which passed off during the night in a sweating stage. On this day, however, she had not been free from fever. Her aspect was languid and rather pale; skin dry; tongue, which was large and indented around the edges, thickly coated; pulse 120; temperature 105°. Had nausea, headache, and backache across the loins; urine scanty and high colored. She was ordered to have twenty grains of quinia sulph. in four doses two hours apart, and to have hot lemonade. On the following morning, at nine o'clock, I found her aspect very much the same. Skin cool and moist; temperature 101°; pulse 70, intermittent, of medium volume, and very compressible, almost gaseous. I was informed that about two hours previous to my visit, without having been in the upright position she had had something like a "fainting spell," which passed away in a few minutes. While I was at the bedside, I observed some indications of distress, which were followed immediately by slight convulsive movement, the head being gradually drawn back, the eyes fixed, the pupils rapidly dilating, and respiration, after one or two spasmodic efforts suspended. All muscular tension was relaxed; the face became mottled from stasis of blood. I had immediately placed my finger upon the radial artery, when, finding no pulsation, I applied my ear over the heart, which gave no throb; meantime the dashing of water, rubbing with camphor and other restoratives had been applied without the slightest effect, and to all appearances she was hopelessly gone.

By the aid of an assistant, I instituted artificial breathing, and kept it up for between five and ten minutes, when I was rewarded by a spasmodic gasp, and by the continued aid of artificial respiration she was gradually resuscitated. She had passed no urine since the evening before; and now, suspecting renal complication, I removed from her bladder less than two ounces (all it contained) of deep amber-colored urine for examination. I may state here that she had no previous history of renal trouble. I ordered five grains quinia sulph. with capsic. every two hours; and from a neighboring drug store I summoned Drs. Wm. Bailey and E. D. Foree, who agreed with me that this and those of the children were cases of profound malarial poisoning.

At this date the boy had fairly entered upon convalescence; nevertheless I had provided myself with a specimen of his urine for examination also. Dr. Baily returned with me to my office for the purpose of examining the specimens, I have predicted the result, which was as follows: Mother's urine—Acidity normal; spec. gravity, 1.012; albumen, twenty-five per cent. Under the microscope it was seen to contain a large number of granular and hyaline casts, together with some free granular matter. Boy's urine—Acidity normal; spec. gravity, 1.020; albumen, a mere trace. The microscope revealed a few hyaline casts, many of them containing more or less granular matter and an occasional granular cast.

At my afternoon visit, ordered three drachms infusion digitalis with twelve grains potasso acetas every three hours.

Without going into further details, I will merely add that the mother had a few hours later an attack similar (though much lighter) to the one I saw her in. She had an evening exascerbation and morning remission for two days, when the temperature became normal; the albumen and tube-casts gradually disappearing, the last specimen containing no granular casts, but an occasional one of purely hyaline character.

As stated above, I have met with these renal manifestations in a number of cases similar to the above; also this summer and fall exactly similar manifestations (except in four of the cases there was complete suppression for periods varying from twenty-four to forty-eight hours), in five cases of what Lebert calls cholera nostra, or sporadic cholera, which I believe to be due also primarily to profound malarial poisoning.

I am well aware that in typical cases of pernicious intermittent and remittent fevers albumen and tube-casts have been occasionally found and mentioned, but I am aware that this complication has been demonstrated in the class of cases above referred to. I wish, therefore, to express the opinion that the condition of the kidneys shown in these cases is far more common than has heretofore been suspected, and constitutes one of the chiefest sources of danger in malarial diseases.—*Louisville Medical News.*

